# Crystal and Electronic Structures of New Two Dimensional 3-NH_3_-PyPbX_4_ Haloplumbate Materials

**DOI:** 10.3390/ma16010353

**Published:** 2022-12-30

**Authors:** Nikita Selivanov, Ruslan Kevorkyants, Alexei Emeline, Constantinos C. Stoumpos

**Affiliations:** 1 Laboratory Photonics of Crystals, Saint-Petersburg State University, 199034 Saint-Petersburg, Russia; 2 University of Hong Kong, Hong Kong, China; 3 Department of Metarials Science and Technology, University of Crete, 71003 Heraklion, Greece

**Keywords:** hybrid haloplumpates, crystal structure, electronic structure, absorption spectra, luminescence spectra, photoexcitation, exciton, charge transfer

## Abstract

In this study, we explored both the crystal and electronic structures of new synthesized materials 3-NH_3_-PyPbX_4_ (X = Br, I). Both compounds are isostructural, and they crystallize in the monoclinic space group *P*2_1_/*c*, with four formula units in the unit cell. According to the analysis of their electronic structures, both compounds are direct semiconductors with direct transitions between valence and conduction bands occurring at the *k*-points A, Z, and at about half of the distance between the *k*-points D/D1 and D1/E. An inspection of DOS reveals that, in both perovskites, the highest energy VBs are comprised mainly of electronic states of halogen anions, while the lowest states in the conduction band originate from lead orbitals. In addition, there are two flat bands composed of electronic states of carbon and nitrogen originating from the organic subsystems and presumably corresponding to the π* orbitals of 3-NH_3_-C_5_H_6_N cations. Both materials demonstrate a broad luminescence emission. Two mechanisms of the radiative relaxation based on either self-trapped excitons or on charge transfers between inorganic and organic subsystems are discussed.

## 1. Introduction

Hybrid lead halide perovskites are an extensive class of photoactive semiconductors, which demonstrate their great potential for various applications in the areas of photovoltaics, photonics, and optoelectronics as light absorbers in solar cells [1], active media for lasers [2], light–emitting materials in LEDs [3,4], and X-ray and gamma radiation detectors [5]. To date, the most studied and most commonly used are 3D perovskites with a general formula APbX_3_ (where A = Cs^+^, CH_3_NH_3_^+^, HC(NH_2_)^+^ and X = Cl^−^, Br^−^, I^−^). At the same time, an increasing interest is devoted to low-dimensional 2D perovskites, which complement and expand the possibilities of perovskite materials due to the possibility of adjusting the crystal structure and the band gap of materials [6].

One of the most promising directions for the application of low–dimensional 2D perovskites is their use as white light sources emitting light in the entire visible spectral range. White light sources based on a single light-emitting material have undeniable advantages over sources using combined radiation from several phosphors to cover the entire visible range since, in the latter case, the light sources can change the color of radiation over the time due to the unequal rate of the degradation of individual light–emitting components, and also are less effective due to the overlapping absorption and emission spectra of phosphors [7].

Most of the currently known perovskite compounds emitting white light belong to the class of low-dimensional 2D organo-inorganic perovskites [7]. Such perovskites consist of separate layers of inorganic anionic octahedra [PbX_6_]^4−^ (where X is the halogen anion), which are separated by large size organic cations (spacers). The studies of the mechanism of the white light emission by perovskites demonstrate that compounds, whose inorganic octahedra experience the strong distortion, are most prone to broadband radiation [8]. In turn, the strong distortion of the octahedra order in the crystal lattice promotes the autolocalization of excitons, whose relaxation results in broadband radiation due to the amplified exciton–phonon interaction [8,9]. Note that the greatest distortion of octahedra is observed in (110) oriented perovskites [8,9,10,11], which are relatively rare compounds. Lattice distortion in (110) oriented perovskites occurs due to the necessity for the efficient packing of organic cations in the cavities between the inorganic layers and the softness of the inorganic halide framework. An appropriate choice of organic cation is a crucial condition for the synthesis of such perovskite compounds capable of emitting the white light. In most cases, the organic cations for such perovskites are protonated diamines. At the moment, about a dozen diamine molecules are known and, with their participation, it was possible to obtain perovskites emitting in a wide range of wavelengths [7]. All of them are aliphatic diamines having substituents in nitrogen atoms. Despite the emerging progress in this field of research, it remains an urgent task to expand the range of organic diamines that can be used for the synthesis of new low–dimensional perovskites with a strongly distorted crystal lattice, which are capable of emitting light in a wide visible spectral range, and to clarify the effect of the crystal structure distortion on the luminescence properties of low dimensional halide perovskites.

Here we report the synthesis of two new (110) oriented 2D perovskites 3-NH_3_-PyPbX_4_ (where X is the halogen anion Br^−^, I^−^) and the results of the exploration of their crystal structure and their electronic and optical properties.

## 2. Experimental

### 2.1. Reagents

Lead (II) bromide PbBr_2_ (98%, Sigma-Aldrich), lead (II) iodide PbI_2_ (99%, Sigma-Aldrich), hydrobromic acid HBr (40% in H_2_O, Iodobrom), hydroiodic acid HI (56% in H_2_O, Iodobrom), hypophosphorous acid H_3_PO_2_ (50% in H_2_O, Acros Organics), and 3-aminopyridine C_5_H_6_N_2_ (99%, Sigma-Aldrich) were used as received. Silica gel was prepared from the sodium metasilicate crystallohydrate solution Na_2_SiO_3_·9H_2_O with the distilled water as a solvent.

To stabilize the hydroiodic acid, hypophosphorous acid was added to it in a 9:1 volume ratio. All solutions and sols, where HI was used as a solvent or reagent, were prepared using the stabilized solution with H_3_PO_2_.

### 2.2. Crystal Growth

**Figure 1:**
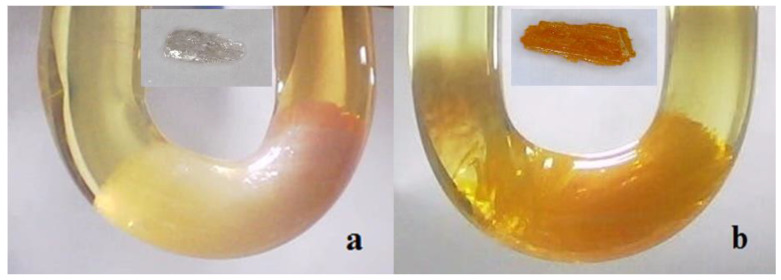
Photo-images of organic-inorganic single crystals grown in silica gel: 3-NH_3_-C_5_H_6_N-PbBr_4_ (**a**) and 3-NH_3_-C_5_H_6_N-PbI_4_ (**b**).

To synthesize the studied compounds, we used the earlier developed method of perovskite single crystal growth in silica gel (CGC method) [12]. This method is based on the difference in solubility between the synthesized perovskite and lead halide (PbX_2_) in the corresponding hydrogen-halide acid (HX). The growth of single crystals occurs in the glass U-tubes filled with silica gel and feeding solutions of lead halide and 3-aminopyridine in hydrogen halogenic acid (Figure 1) with concentrations of 1 M poured over silica gel.

### 2.3. Structural Characterization

A single crystal X-ray diffraction study of the synthesized single crystals was carried out using diffractometer Agilent Technologies Xcalibur (Oxford Diffraction). XRD measurements were performed at a temperature of 100 K. 1. Data collection and data reduction were performed using a ChrysalisPro (ver. 1.171.40.71a) using either a Mo Kα source (Xcalibur EoS 4-cycle diffractometer equipped with a CCD detector) or a Cu Kα source (XtaLAB Synergy 4-cycle diffractometer equipped with a HyPix CCD detector). A numerical absorption correction was applied using the build in the ABS PACK algorithm. Crystal structures were solved and, by charge flipping, refined (full-matrix least-squares on F^2^) using the Jana2006 package [13].

Powder X-ray diffraction studies were conducted with the X-ray diffractometer Bruker “D2 Phaser” using an X-ray tube Cu Kα anode. Reflected X-rays were detected using a solid position sensitive detector LYNXEYE. Measurements were conducted at room temperature. Pattern matching was performed using the Jana2006 package [13].

### 2.4. X-ray Photoelectron Spectroscopy

The X-ray photoelectron spectroscopy was accomplished with the Thermo Fisher Scientific ESCALAB 250Xi X-ray photoelectron spectroscopy system with an Al-Kα X-ray source (1486.6 eV with a monochromator) and the tunable beam’s size (200–900 μm) on a sample’s surface. The energy resolution was 0.45 eV.

### 2.5. Computational Approach

DFT computations with periodic boundary conditions were conducted using the VASP 5.4.4 program [14,15,16,17]. Electronic structures were modeled using the plane augmented wave (PAW) approach [18] in conjunction with PBE [19] density functional. The SCF cutoff was set to 1.0 × 10^−7^ eV. Electronic band structures were plotted along the Γ-A-C-D-D1-E-Y-Y1-Z path of a monoclinic Brillouin zone. Chemical structures were visualized using the program VESTA [20], whereas electronic band diagrams were plotted using the software package Gnuplot 5.2.

### 2.6. Diffuse Reflectance Spectroscopy

The diffuse reflectance spectroscopic studies of the powdered samples were conducted using a Cary 5000 UV-Vis-NIR spectrometer equipped with a diffuse reflectance accessory DRA 2500. Barium sulphate was used as a reference sample. Powders were prepared by grinding single crystals in an agate mortar.

### 2.7. Photoluminescence Spectroscopy

Photoluminescence and excitation spectra were recorded at the temperature of 77 K and at room temperature using the spectrofluorometer Agilent Cary Eclipse.

## 3. Results and Discussion

### 3.1. Crystal Structure

**Figure 2:**
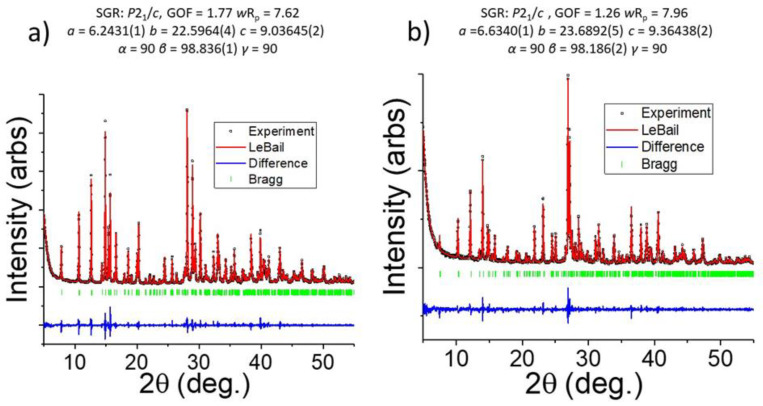
Experimental powder XRD patterns (Cu Kα (λ = 1.5406 Å) of 3-NH_3_-PyPbX_4_ (**a**) X = Br; (**b**) X = I) and their corresponding LeBail fitting at room temperature. The pattern matching reveals no change in the space group between the 100 K structures obtained from single-crystals and 293K. The obtained unit cell parameters show a significant thermal expansion which is expected for 2D perovskites.

Aminopyridinium-based perovskites were prepared in a chemically pure form using the silica gel slow diffusion method in U-shaped tubes. The compounds 3-NH_3_PyPbX_4_ (X = Br, I) were the only observable product in the reaction system having a 1:1 stoichiometry for the reactant cations (halogens were used in excess). The chemical purity of the materials was examined with powder X-ray diffraction at room temperature. Pattern matching of the two compounds based on the single-crystal data models (see below) were performed, resulting in an excellent fitting in the monoclinic space group *P*2_1_/*c*. The refinement results ([20], Figure 2) indicate that there is no significant change in the materials between 100 K and room temperature, exhibiting only a drastic thermal expansion in all crystallographic directions. 

**Figure 3:**
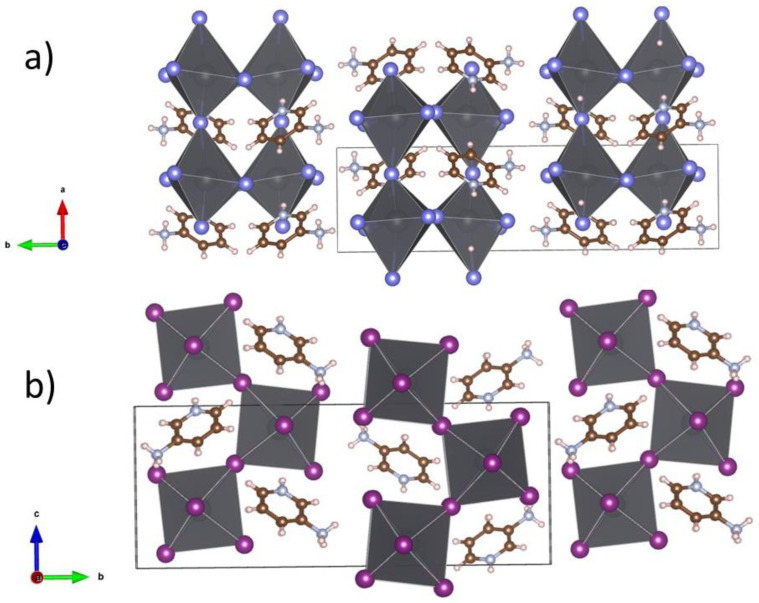
Crystal structures of the isostructural compounds 3-NH_3_-PyPbX_4_ ((**a**) X = Br; (**b**) X = I) at 100 K. 3-NH_3_-PyPbBr_4_ is represented along the crystallographic *c*-axis and 3-NH_3_-PyPbI_4_ along the crystallographic *a*-axis. Color code: Pb gray; I purple; Br blue; C brown; N light blue; H pink.

The crystal structure of the two compounds was determined by single-crystal diffraction experiments at 100 K. The structures were solved and refined using the Jana2006 software package [13]. Both compounds are isostructural, and they crystallize in the monoclinic space group *P*2_1_/*c*, with four formula units in the unit cell. Crystallographic data are given in Table 1, while structural models are plotted in Figure 3.

3-NH_3_-PyPbX_4_ compounds consist of corrugated (PbX_4_)^2−^ monolayers belonging to the (110)-sliced family of halide perovskites. The structure is stabilized by intercalating aminopyridinium cations which act as spacers between the perovskite sheets. The nitrogen atoms of aminopyridine are fully protonated, resulting in a divalent aminopyridinium cation. Unlike the crystal structure of most (110)-sliced perovskites [9,11,21,22,23,24,25], the ammonium group points at the interlayer space between adjacent perovskite sheets and, instead, it is the ammonium part of the cation that occupies the perovskite “pocket”. As a result, the octahedra undergo a large axial distortion along the crystallographic a-axis (Figure 3a), influenced by strong intermolecular forces between the inorganic lattice and the organic spacer. Further, the perovskite distortion seems to adopt the aminopyridinium shape and charge distribution, producing close contact between the hydrogen bonded (−NH_3_^+^ group) inorganic layers. 

### 3.2. Electronic Structure

**Figure 4:**
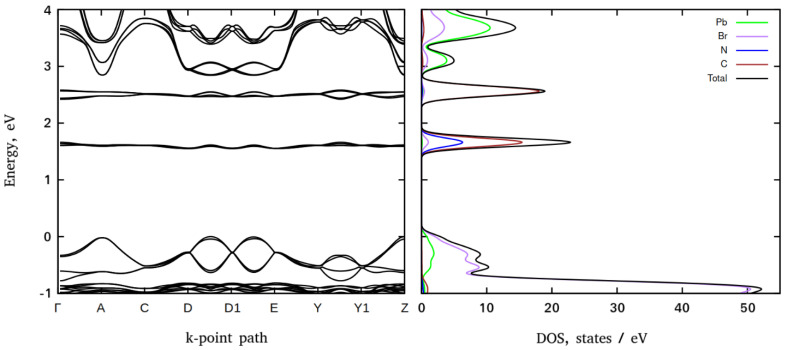
PBE electronic BS (**left**) and DOS (**right**) of 3-metaaminopyridinium lead bromide.

**Figure 5:**
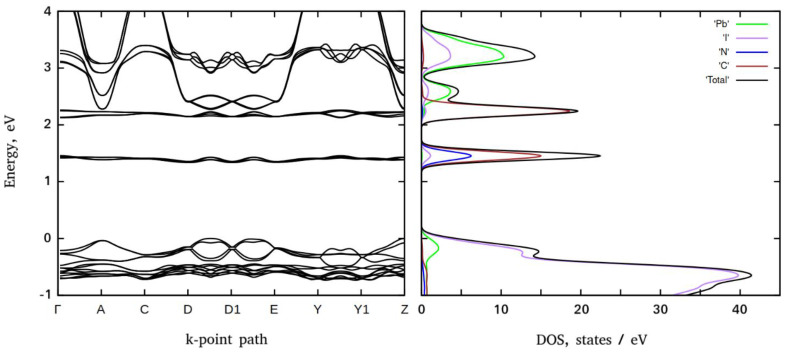
PBE electronic BS (**left**) and DOS (**right**) of 3-metaaminopyridinium lead iodide.

The electronic band structures (BS) and density of states (DOS) of the 3-NH_3_-C_5_H_6_N-PbBr_4_ and 3-NH_3_-C_5_H_6_N-PbI_4_ perovskites are presented in Figure 4 and Figure 5, respectively.

Both perovskites feature the same structure of the primitive cells and mutual orientation of the inorganic and organic subsystems. This results in very similar band structures. In both compounds the highest valence band (VB) energies occur at the *k*-points A, Z, and at about half of the distance between the *k*-points D/D1 and D1/E. An inspection of DOS reveals that, in both perovskites, the highest energy VBs are comprised mainly of the electronic states of halogen anions. The lowest conduction band (CB) energies reproduce the same trend and occur at *k*-points A, Z, and at about half of the distance between the *k*-points D/D1 and D1/E. According to DOS data, the major contribution in the conduction band states originates from lead orbitals. Thus, electronic excitations from VB to CB shall take place between these *k*-points and infer that both perovskites are direct semiconductors. The computed bandgaps corresponding to the energy difference between the top of the valence band and the bottom of the conduction band formed by the electronic states of inorganic subsystems are predicted to be ~2.84 eV (bromide) and ~2.27 eV (iodide) at the Z-point that follows the typical trend in the family of halide perovskites, namely, given that the same crystal symmetry iodide perovskites demonstrate smaller bandgaps than the bromide ones. 

In addition to the VB and CB states, in the electronic structure of both compounds, there are two flat bands composed of electronic states of carbon and nitrogen (~1.60 eV and ~2.50 eV for bromide and ~1.40 eV and ~2.20 eV for iodide perovskites, respectively). The ratio of carbon to nitrogen states is approximately equal to the ratio of carbon to nitrogen atoms in the organic subsystem. Thus, they originate from the organic subsystems and presumably correspond to the π* orbitals of 3-NH_3_-C_5_H_6_N cations. As expected, the probabilities of electronic transitions from the inorganic subsystems to the organic ones are rather low compared to the probabilities of VB to CB electronic transitions. 

**Figure 6:**
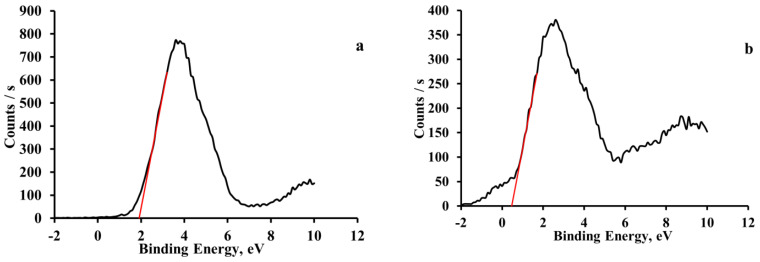
XPS spectra of the electronic states corresponding to the valence bands of the studied compounds: 3-NH_3_-PyPbBr_4_ (**a**), and 3-NH_3_-PyPbI_4_ (**b**).

The positions of the top states of the valence bands (in the vacuum energy scale) were estimated experimentally by the measurements of the XPS spectra in the energy range corresponding to the valence bands of the compounds (see Figure 6).

An estimation of the energy of the top of the valence band was performed by drawing a tangent to the intersection with the x-axis at the low-energy edge of the XPS spectra. The estimated energy values of the top states of the valence bands corrected to the Au work function where the vacuum energy level are −1.9 eV for 3-NH_3_-C_5_H_6_NPbBr_4_ and −0.5 eV for 3-NH_3_-C_5_H_6_NPbI_4_, with respect to the Fermi level.

### 3.3. Absorption and Luminescence

**Figure 7:**
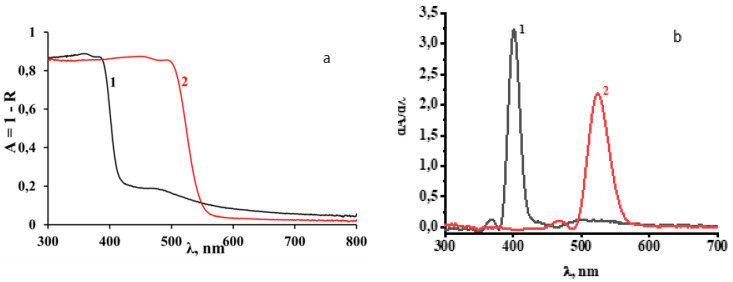
Absorption spectra (**a**) and their first derivatives (**b**) of 3-NH_3_-PyPbBr_4_ (1) and 3-NH_3_-PyPbI_4_ (2).

The absorption spectra have been obtained from the diffuse reflectance spectra (A = 1 − R, where R is the diffuse reflection coefficient) and are shown in Figure 7.

The absorption spectrum edge of 3-NH_3_-PyPbI_4_ is shifted by about 100 nm to the longer wavelength spectral region compared to the absorption spectrum of 3-NH_3_-PyPbBr_4_. Two regions can be distinguished in the absorption spectra of the studied compounds: the shorter wavelength corresponding to the strong fundamental absorption and the longer wavelength spectral regions. The shorter wavelength regions with an edge at 400 nm for 3-NH_3_-PyPbBr_4_ and 500 nm for 3-NH_3_-PyPbI_4_, respectively, are formed by electronic transitions from the filled p orbitals of halogen anions to vacant p orbitals of lead and halogen ions in inorganic layers consisting of perovskite octahedra [PbX_6_]^4-^ (see Figure 4 and Figure 5). The application of the spectra analysis using their first derivatives demonstrates that the strongest alteration of the sample absorptions corresponding to the fundamental absorption edges occur at 400 nm (~3.1 eV) and 525 nm (~2.36 eV), with minor features at shorter wavelengths at 370 nm (~3.35 eV) and 470 nm (~2.64 eV) for 3-NH_3_-PyPbBr_4_ and 3-NH_3_-PyPbI_4_, respectively. The longer wavelength region in the absorption spectrum of these compounds, we believe, may be associated with electronic transitions between inorganic layers and manifest π* states of the organic 3-aminopyridinium cations [26]. Particularly, it manifests itself as a minor first derivative feature at 500 nm (~2.5 eV) observed for 3-NH_3_-PyPbBr_4_.

**Figure 8:**
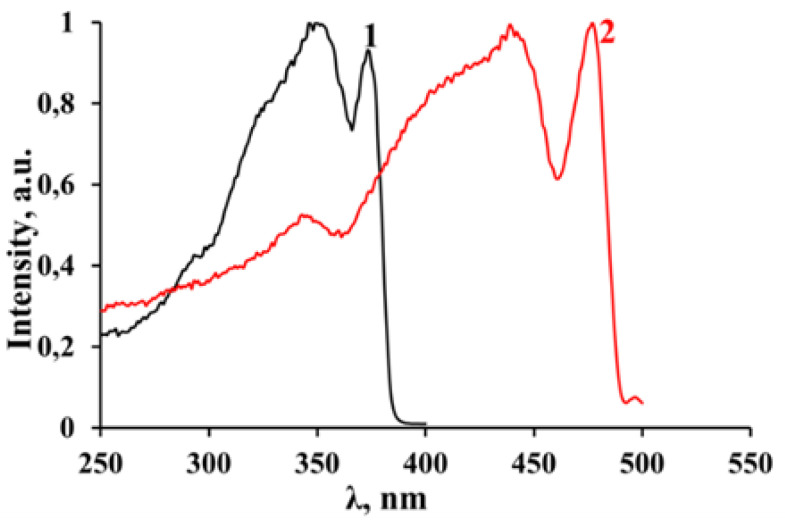
Excitation spectra of photoluminescence of 3-NH_3_-PyPbBr_4_ (1) and 3-NH_3_-PyPbI_4_ (2) recorded at T = 77 K.

The excitation spectra of luminescence and the luminescence spectra of the studied compounds were recorded at 77 K (Figure 8). The excitation spectra of luminescence (Figure 3), with an emission registration at the wavelengths corresponding to the maxima of the luminescence spectra, consist of several resolved bands with clearly distinguishable maxima: in the region of 250 nm–400 nm with a threshold at 390 nm and the maxima at 375 nm (~3.3 eV) and 340 nm (~3.65 eV) for the perovskite with bromine anions, and in the region of 250 nm–500 nm with a threshold at 490 nm and the maxima at 475 nm (~2.6 eV) and 440 nm (~2.8 eV) for the perovskite with iodine anions. 

A comparison of these spectra with the absorption spectra shows that, in general, the edge of the excitation spectra coincides well with the edge of the shorter wavelength absorption region of perovskites attributed to the excitation of inorganic layers consisting of perovskite octahedra. However, the obvious “red” shift of the absorption features (see Figure 7) recorded at room temperature comparing to the excitation spectra of the studied samples recorded at 77 K indicates a possibility of a temperature dependence of the band gap energy. Presumably, the longer wavelength excitation maxima (375 nm and 475 nm) in both compounds might be attributed to absorption, resulting in the generation of free exciton (FE) states, while the excitation at shorter wavelengths corresponds to electronic transitions from VB to CB. 

**Figure 9:**
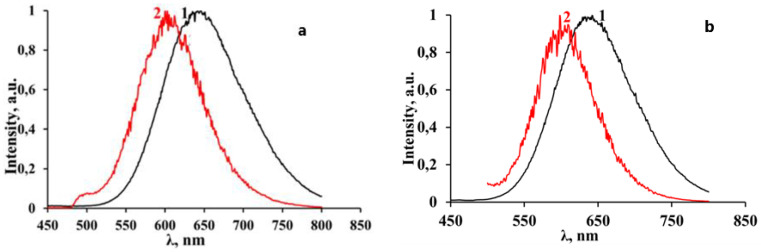
Photoluminescence spectra of 3-NH_3_-PyPbBr_4_ (**a**) and 3-NH_3_-PyPbI_4_ (**b**) recorded at 77 K with excitation wavelengths: (**a**) 340 nm and 440 nm and (**b**) 375 nm and 475 nm, respectively.

The luminescence spectra of both 2D perovskites consist of broad bands in the visible region with maxima at 600 and 640 nm for compounds with iodide and bromide anions, respectively. The spectral shapes of the luminescence bands do not depend on the excitation wavelengths for both compounds (see Figure 9). 

The luminescence spectrum of 3-NH_3_-PyPbI_4_, in contrast to 3-NH_3_-PyPbBr_4_, also demonstrates the second luminescence band of lower intensity with a maximum at around 500 nm. This band in low-dimensional perovskites is usually attributed to a free exciton (FE) emission [24,27]. An alternative explanation of the origin of this luminescence band could be an overlapping of CB states with π* orbitals of the 3-NH_3_-Py cation that results in a charge transfer from an inorganic to organic subsystem, followed by radiative relaxation to the ground state (back charge transfer to inorganic VB states) of perovskite. The studied two-dimensional perovskites, as well as the majority of low-dimensional perovskite compounds, are characterized by a significant Stokes shift of the luminescence spectra. The luminescence spectrum of 3-NH_3_-PyPbBr_4_ shows a much larger Stokes shift compared to the spectrum of 3-NH_3_-PyPbI_4_. The large Stokes shift of the luminescence indicates a strong electron-phonon interaction in the excited state; the corresponding emission may be a consequence of the relaxation of self-trapped excitons (STE) [24,27]. Accordingly, the excitation of the observed luminescence corresponding to the radiative relaxation of STE states can be attributed to the same band to band electronic transitions in inorganic parts of the perovskite structures, followed by the formation of the FE states with their consequent self-trapping.

**Figure 10:**
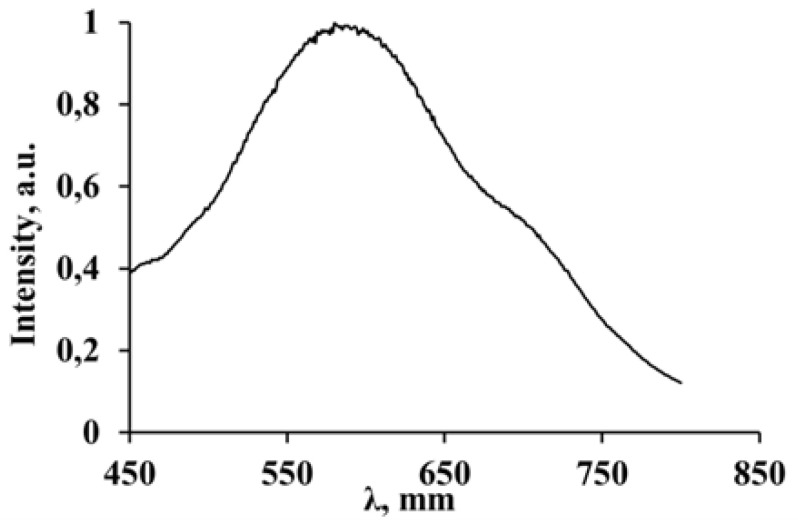
Luminescence spectrum of 3-NH_3_-PyPbBr_4_ registered at room temperature (λ_exc._ = 440 nm).

At room temperature, 3-NH_3_-PyPbB_4_also demonstrates quite intense luminescence (Figure 10).

**Figure 11:**
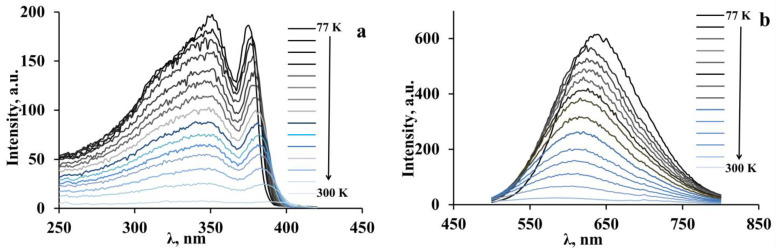
Temperature evolution (77–295 K) of the excitation spectra (**a**) and luminescence spectra (**b**) of 3-NH_3_-PyPbBr_4_. The maximal luminescence intensity corresponds to 77 K.

**Figure 12:**
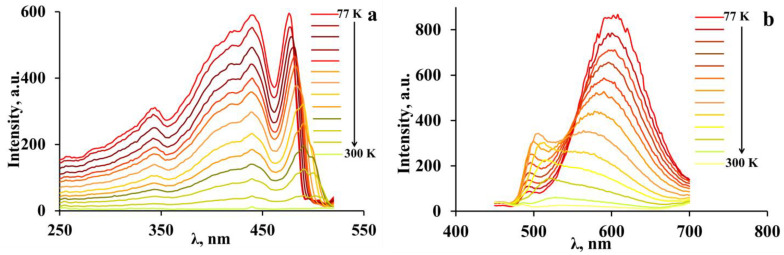
Temperature evolution (77–295 K) of the excitation spectra (**a**) and luminescence spectra (**b**) of 3-NH_3_-PyPbI_4_. The maximal luminescence intensity corresponds to 77 K.

In addition, we investigated the effect of the temperature on the behavior of the luminescence spectra and excitation spectra of both perovskites caused by the uncontrolled heating of the samples from 77 K to room temperature (Figure 11 and Figure 12).

The luminescence spectra of 3-NH_3_-PyPbBr_4_ show a decrease in the emission intensity and a gradual hypsochromic shift of 55 nm of the luminescence band maxima upon heating (Figure 11b). A similar luminescence maxima shift during heating/cooling is a characteristic of other low-dimensional perovskite compounds [8,9]. This shift may be a consequence of an increase in the anharmonic vibrations of the crystal lattice with a temperature increase [10]. Such behavior of the luminescence spectra is also observed for the excitation spectra of the luminescence of 3-NH_3_-PyPbBr_4_. At the same time, the long wavelength edge of the excitation band demonstrates a bathochromic shift of up to 10 nm, which may be associated with a decrease in the band gap width due to thermal effects, such as lattice expansion with temperature increasing and an alteration in phonon distribution [10].

The longer wavelength band in the luminescence spectra of 3-NH_3_-PyPbI_4_ is also characterized by a decay in luminescence intensity and a hypsochromic shift of the emission maximum (Figure 12b). In contrast, the short wavelength band with the luminescence maximum near 500 nm grows in intensity and experiences a bathochromic shift. Thus, there is a tendency to combine these two bands into one band with a blurred maximum, whose intensity decreases rapidly with increasing temperature. 

The luminescence excitation spectra of 3-NH_3_-PyPbI_4_ with increasing temperature (figure) demonstrates a bathochromic shift of the long wavelength edge by 15 nm, and the corresponding band is broadening, which may also be caused by the lattice expansion mentioned above [10].

The analysis of the obtained experimental results of excitation and luminescence processes infers that two different mechanisms can be considered (Table 2).

Excitation processes in both mechanisms are the same and consist of photon absorption, leading to either band to band transitions resulting in the generation of free charge carriers or to excitonic absorption. At 77 K, free charge carriers can form free excitons as an intermediate state of excitation. Mechanism I describes the relaxation processes as either the relaxation of free excitons responsible for the short wavelength luminescence at 500 nm in 3-NH_3_-PyPbI_4_ or the exciton self-trapping (STE), yielding broad band luminescence in both perovskites. Alternatively, mechanism II describes the short wavelength luminescence at 500 nm in 3-NH_3_-PyPbI_4_ as a charge transfer (CT) of electrons from the conduction band to the π* orbitals of 3-NH_3_-Py cations, followed by radiative back CT from π* orbitals to valence band. Note that the latter mechanism is supported by theoretical results demonstrating that the highest states of π* orbitals are located close to the bottom states of the conduction band, and temperature induced blurring of the CB edge may significantly improve the overlapping of CB states and π* orbitals and, therefore, promote the CT process. Note that this CT mechanism may be not valid for 3-NH_3_-PyPbBr_4_ where, according to the theoretical modeling of the electronic structure, such overlapping between CB states and π* orbitals is unlikely to happen.

## 4. Conclusions

Two new 2D dimensional perovskites, 3-NH_3_-PyPbB_4_ and 3-NH_3_-PyPbI_4_, were synthesized. Both adopt the (110)-sliced perovskite structure, exhibiting strong intermolecular interactions between the inorganic and organic components. Both compounds demonstrate broad band luminescence in the visible range at 77 K, and 3-NH_3_-PyPbBr_4_ retains the luminescence ability, even at room temperature. The mechanism of luminescence is based on the formation and relaxation of STE states. At the same time, the CT mechanism involving π* orbitals of 3-NH_3_-Py cations may also be responsible for the short wavelength luminescence of 3-NH_3_-PyPbI_4_.

## Figures and Tables

**Table 1:** Crystal data and structure refinement for 3-NH_3_-PyPbX_4_ at 100 K.

3-NH_3_-PyPbX_4_	3-NH_3_-PyPbBr_4_	3-NH_3_-PyPbI_4_
Formula weight	622.9	810.9
Temperature	100 K	
Wavelength	1.54184 Å	0.71073 Å
Crystal system	monoclinic	
Space group	*P*2_1_/*c*	
Unit cell dimensions	a = 6.21010(10) Å, α = 90° b = 22.4384(3) Å, β = 99.3209(11)° c = 8.96470(10) Å, γ = 90°	a = 6.5996(2) Å, α = 90° b = 23.5079(11) Å, β = 98.548(4)° c = 9.2898(5) Å, γ = 90°
Volume	1232.69(3) Å^3^	1425.24(11) Å^3^
Z	4	4
Density (calculated)	3.3566 g/cm^3^	3.7793 g/cm^3^
Absorption coefficient	41.471 mm^−1^	20.466 mm^−1^
F(000)	1096	1384
θ range for data collection	3.94 to 77.56°	2.81 to 34.84°
Index ranges	−7 ≤ h ≤ 7, −28 ≤ k ≤ 28, −11 ≤ l ≤ 11	−10 ≤ h ≤ 9, −36 ≤ k ≤ 36, −14 ≤ l ≤ 14
Reflections collected	20,093	12,882
Independent reflections	2600 [R_int_ = 0.0986]	5735 [R_int_ = 0.0339]
Completeness to θ	99%	98%
Refinement method	Full-matrix least-squares on F^2^	
Data/restraints/parameters	2600/0/109	5735/0/109
Goodness-of-fit	3.41	1.37
Final R indices [I > 2σ(I)]	R_obs_ = 0.0570, wR_obs_ = 0.1260	R_obs_ = 0.0380, wR_obs_ = 0.0781
R indices [all data]	R_all_ = 0.0573, wR_all_ = 0.1261	R_all_ = 0.0437, wR_all_ = 0.0799
Largest diff. peak and hole	4.58 and −2.11 e·Å^−3^	1.70 and −3.64 e·Å^−3^

R = Σ||F_o_| − |F_c_||/Σ|F_o_|, wR = {Σ[w(|F_o_|^2^ − |F_c_|^2^)^2^]/Σ[w(|F_o_|^4^)]}^1/2^ and w = 1/(σ^2^(I) + 0.0004I^2^).

**Table 2:** Two possible mechanisms of radiative relaxation (luminescence).

Steps of the Processes	Mechanism I	Mechanism II
Excitation	3-NH_3_-PyPbX_4_ + hν → e + h 3-NH_3_-PyPbX_4_ + hν → e^0^ (FE) e + h → e^0^ (FE)	
Relaxation	e^0^(FE) → hν e^0^(FE) → STE → hν	e^0^(FE) + π* → π*(e) (CT) e + π* → π*(e) (CT) π*(e) → hν

## Data Availability

Not applicable.
